# Novel Technologies in Studying Brain Immune Response

**DOI:** 10.1155/2021/6694566

**Published:** 2021-03-18

**Authors:** Li Li, Cameron Lenahan, Zhihui Liao, Jingdong Ke, Xiuliang Li, Fushan Xue, John H. Zhang

**Affiliations:** ^1^Department of Anesthesiology, Beijing Friendship Hospital, Capital Medical University, Beijing 100053, China; ^2^Burrell College of Osteopathic Medicine, Las Cruces, NM 88003, USA; ^3^Center for Neuroscience Research, School of Medicine, Loma Linda University, Loma Linda, CA 92324, USA; ^4^Department of Anesthesiology, Neurosurgery and Neurology, School of Medicine, Loma Linda University, Loma Linda, CA 92324, USA; ^5^Department of Physiology and Pharmacology, Basic Sciences, School of Medicine, Loma Linda University, Loma Linda, CA 92324, USA

## Abstract

Over the past few decades, the immune system, including both the adaptive and innate immune systems, proved to be essential and critical to brain damage and recovery in the pathogenesis of several diseases, opening a new avenue for developing new immunomodulatory therapies and novel treatments for many neurological diseases. However, due to the specificity and structural complexity of the central nervous system (CNS), and the limit of the related technologies, the biology of the immune response in the brain is still poorly understood. Here, we discuss the application of novel technologies in studying the brain immune response, including single-cell RNA analysis, cytometry by time-of-flight, and whole-genome transcriptomic and proteomic analysis. We believe that advancements in technology related to immune research will provide an optimistic future for brain repair.

## 1. Introduction

The role of the immune system in brain function and development has been highlighted by several studies [1–3]. Immunomodulation has even been considered a potential therapeutic strategy for neurodegenerative dysfunctions, including Alzheimer's disease, stroke, and other neurodegenerative conditions [4].

Immune cells are complex and dynamic and characterized by diverse cell profiles. Besides, their activity largely relies on their interactions with each other, along with other pathological processes following brain injury [5, 6]. As a result, to obtain a comprehensive knowledge of the brain immune system, detailed profile information of each cell is required. The progress of human science and technology, especially the establishment and improvement of RNA sequencing, mass cytometry, and whole-genome transcriptomic and proteomic analysis, allows us to delve deeper and wider into how the brain immune system functions and responds to various pathophysiological conditions. In these techniques, RNA sequencing methods have assisted in depicting the complexity and diversity of the examined immune cells [7]. Cytometry by time-of-flight (CyTOF) overcomes the limitations of traditional flow cytometry approaches to analyze more than 20 parameters, allowing us to detect single-cell protein information, which is vital for revealing cell functions [8]. The focus of whole-genome transcriptomic and proteomic analysis is to chart and map cell information at DNA, RNA, and protein levels, plotting a much deeper, uncharted territory for future neurobiologists. In this paper, we will review the use of these technologies in the brain's immune system. The literature was searched using Medline between 2006 and the present using search terms including “single-cell RNA”, “Cytometry by time-of-flight”, “whole-genome transcriptomic and proteomic analysis”, and “immune response”. The search terms were cross-referenced, and the search was limited to English language articles. All of the articles, found including those associated with the initial search results, were evaluated for methodology and results and were included if deemed applicable to this review.

## 2. Immune Response in the Brain

The brain was previously thought to be an “immunologically privileged” organ because peripheral immune cells have been considered unable to cross the blood-brain barrier. Now, mounting evidence suggests that glial cells, particularly microglia and astrocytes, constitute and modulate the complex neuroimmune system of the brain in response to infection and drug intervention [1, 2]. As research continues, macrophages, oligodendrocytes, and endothelial cells have also been found to play an important role in the process of neuroimmunity [9]. Activation of these neuroimmune cells may impair healthy neurons and result in brain damage by releasing pro-/anti-inflammatory cytokines and reactive oxygen species (ROS). Notably, this system is also important for brain metabolism and development by regulating synaptogenesis, neurotransmitter transmission, and trophic support for neurons [10, 11]. Therefore, brain immune may play a pivotal role in the pathology of several neurological disorders and the development of the brain. However, the process and mechanism of the neuroimmune system are not completely understood for the structural complexity of the brain. The possibility to elucidate the current neuroimmune state has attracted a remarkable interest of a large number of neuroscientists. New insights in this field will provide novel methods to measure the neuroinflammatory process, facilitate the discovery of potential effective biomarkers, and eventually lead to the alleviation, treatment, and prevention of these nervous system diseases which may stimulate a neuroimmune response.

## 3. Novel Tools in Studying Brain Immune Response

### 3.1. Single-Cell RNA-seq Technology

In recent years, single-cell RNA-seq (scRNA-seq) has become widely used for transcriptome analysis in the field of the immune system. It provides quantitative measurements of RNA molecules within a single cell, leading to the present rapidly expanding world of single-cell transcriptomics. Single-cell genomics makes it possible to create a high-resolution characterization of cells and allows the profiling of numerous molecules from single cells. It is hoped that scRNA-seq will be used to create a comprehensive reference map of the types and properties of all human cells, as a basis for understanding, diagnosing, monitoring, and treating health and disease.

**Figure 1:**
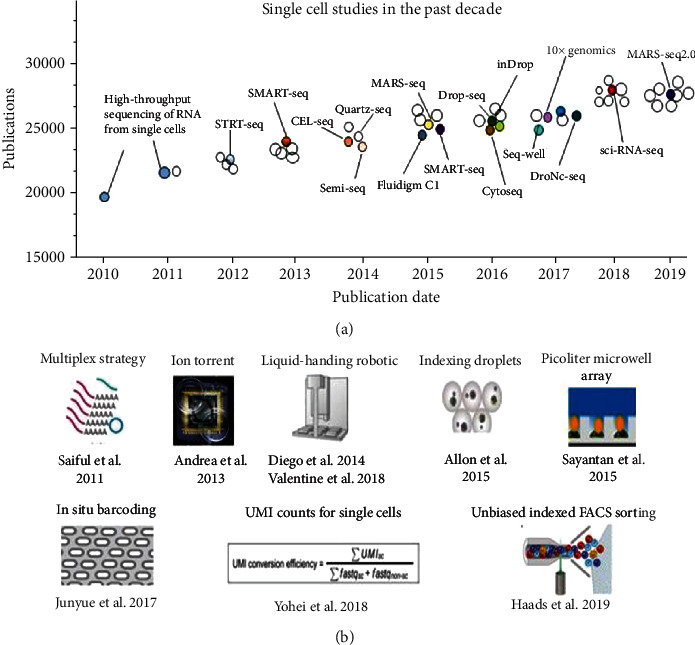
Single-cell studies in the past decade. (a) Single-cell study numbers reported in representative publications in the past decade. Key technologies are indicated; (b) key technologies in cell sorting scale. More than one thousand cells were enabled by large-scale studies using multiplex strategy, followed by a jump to several thousands of cells with liquid-handling robotics. Further, automated massively parallel single-cell RNA sequencing that brought the assayed cell numbers into tens of thousands was enabled by random capture technologies using indexing droplets and picoliter microwell array technologies. Sci-RNA-seq used the split-pool barcoding of nucleic acid method to uniquely label hundreds of thousands of cells. Quartz-Seq2 was developed based on Quartz-Seq, achieving a 30-50% increase in the effectiveness with which the initial sequence reads were converted to the unique molecular identifier (UMI) expression. Recent protocol MARS-seq2.0 combines indexed fluorescence-activated cell sorting (FACS) and robotics with multiple layers of molecular barcoding, by which cell types derived from different tissues and organisms could be located and identified. UMI = unique molecular identifier; FACS = fluorescence-activated cell sorting.

The usage of scRNA-seq has grown exponentially during these years (Figure 1). However, to analyze scRNA-seq data, novel methods are required. There are many scRNA-seq protocols, including CEL-seq2/C1, Drop-seq, MARS-seq [12, 13], SCRB-seq, Smart-seq/C1, Smart-seq2, STRT-seq, inDrop, Semi-seq [14], SPLIT-seq by using SMART-seq2 and plates [15–17], massively parallel single-cell RNA-seq [18, 19], droplets [20], microfluidics [21–23], 10x chromium system [24], sci-RAN-seq [25], Quartz-Seq2 [26], and the recently developed MARS-seq2.0 [27]. Each protocol has individual advantages and limitations, so their respective usage is often dictated by multiple features, such as biological context, cost, objective, and experimental scale [28, 29]. Ziegenhain et al. and Svensson et al. tested and compared the sensitivity and specificity between protocols that are commonly used today, providing a guide for immunologists before choosing an appropriate approach for addressing a particular research problem [29, 30], followed by Haque et al., who give researchers a further practical guide for their first scRNA-seq practice [31] (Table 1).

#### 3.1.1. Characterizing the Diversity within a Population of Brain Immune Cells

The diversity of gene expression may be the key for the immune system to maintain homeostasis and against infections [32]. scRNA-seq provides a new and effective method to uncover the transcriptional patterns within a population of cells. It has been developed and optimized for diversity analysis in many kinds of cells, which is the main reason why immunologists choose scRNA-seq for study. Recently, deep single-cell RNA sequencing has been applied to study the developmental heterogeneity of microglia [33]. The results displayed an obvious difference in the gene expression of microglia between the adult and early postnatal brain by collecting and analyzing samples from different brain regions. These findings are important to show that the heterogeneity may have potential functions in immune cell development and differentiation. Furthermore, scRNA-seq has been used to target the diversity of glial cells and drug-induced neuroinflammation in cerebral organoids [34]. Previous cerebral organoid studies using scRNA-seq primarily focused on neural populations. Dang and his team turned their attention to glial cells; they confirmed the diversity of glial cells at the organoid level and the neuroinflammatory response within drug-treated organoid astrocytes with single-cell resolution.

#### 3.1.2. Future Directions for scRNA-Seq in Brain Immune System

**Figure 2:**
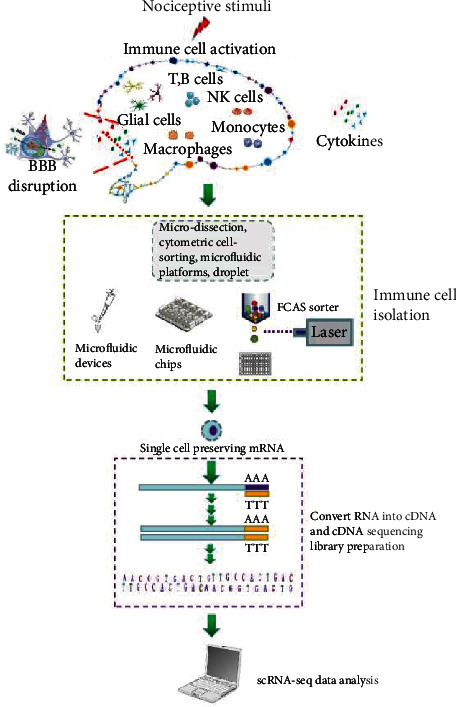
Brain immune cell scRNA-seq workflow. Nociceptive stimuli including infection and drug intervention can cause an activation of glial cells, lymphocytes, monocytes, and neutrophils. Activation of these neuroimmune cells results in pro-/anti-inflammatory cytokine release, blood-brain barrier (BBB) disruption, and subsequent brain damage. Single-cell RNA sequencing technologies using many strategies such as microfluidic devices, fluorescence-activated cell sorting (FACS) into 96-well plates, and microfluidic chips for cell isolation to obtain single-cell preserving mRNA. Then, reverse transcription and PCR amplification are carried out by droplet-based or plate-based approaches. Next, the transcription of primed RNA was converted into complementary DNA (cDNA) using reverse transcription. After this, cDNA amplification was conducted by PCR, and then, the cDNA sequencing library was prepared. Finally, we use specialized software to analyze and present the data.

*(1) Revealing Cell Fate Branching Point*. While cell differentiation, proliferation, and reprogramming are controlled by complicated gene regulatory networks, each cell determines its own destiny by the integration of a large number of signals, which makes the cell fate difficult to be dissected for technology limits. In addition to heterogeneity discrimination, the appearance of single-cell techniques that can simultaneously detect mountains of molecules in a single cell could also shed some light on this challenge. Chen used single-cell profiling strategy to identify the heterogeneity of CD8+ T cells during acute and chronic lymphocytic choriomeningitis virus infections [7]. In this study, CD8+ T cells showed a different fate trajectory responding to the same virus infection. Notably, the distinct transcriptional profiles of CD8+ T cells and the transcriptional bifurcation timing have been determined with scRNA-seq [7]. Except for immune cells, this novel tracing technology also shows promise in other cell types including basal and myoepithelial cell population in mapping the cell fate and branch points [35]. Viewed from this point, scRNA-seq is expected to be used to reveal cell fate branch points of the brain immune system (Figure 2).

*(2) Classification of the Cellular Composition*. Notably, scRNA-seq could provide an unexpected opportunity to systematically identify the cellular specializations of brain cells. To obtain a deeper understanding of the brain, scientists have attempted to establish experimental brain organoid models outside of the context of embryogenesis [36]. Brain organoids originating from different stem cells have been reported from time to time [37, 38], but the significance lies in whether they can generate the similar and rich diversity of cell types appropriate for the brain. This is one of the most important characteristics of the brain that distinguishes it from the rest of the body. However, few studies have attempted to classify the cellular composition of these organoid models, partly due to technical limitations. However, the situation is improving as technology advances. Velasco and her stem cell research team have tested four individual dorsal forebrain organoid models established by four distinct protocols and observed that each model can stably generate a reproducible cellular diversity without the requirement of an embryo [39]. Their important discovery was published in *Nature* recently. This is a typical case where cutting-edge technologies are used to solve the challenges of scientific research. Another important work worth mentioning is the development of a new algorithm called LIGER, which is designed to delineate shared and dataset-specific cell identity features [40]. As a promising analytical tool, LIGER could combine the single-cell RNA-seq information with DNA methylation profile data and credibly clarify the cell type and single-cell gene expression in specific subsets of human and mouse brain cells.

Single-cell sequencing transcriptomics can also be used to trace the origin and distribution schema of some key molecules and cells of the brain, which comprises thousands of cell types [41]. Undoubtedly, it is extremely useful for understanding the development and diseases of the human brain, where so many mysteries remain uncovered. Technological advances in scRNA-seq make it possible for researchers to achieve a detailed cell-type analysis of different cells and tissues. Therefore, we can use this advantage of scRNA-seq to reveal immune cell types involved in a specific brain immune response caused by different causes and finally find a treatment target.

*(3) Revealing the Interaction between Cells*. Apart from the cell-type analysis, scRNA-seq could be used to reveal an interaction between cells. scRNA-seq of the subventricular zone (SVZ) has been separately performed in young (3 months old) and old (28-29 months old) mouse brains to explore how the neurogenic niche, which comprises neural stem cells, as well as many other cell types, changed with age [42]. An expansion of T cells and a decreased proliferation of neural stem cells in old mouse brains were detected in this study. scRNA-seq results indicated that the presence of brain T cells correlated with an inhibition of neural stem cell proliferation, partly because of interferon-*γ* secretion. This gives us a new way to study the interaction between the peripheral and central immune systems.

scRNA-seq could also be used in conjunction with other popular techniques. It was performed on GFP-labeled inhibitory interneurons (INs) to explore how neocortical projection neurons instruct IN circuit development [43]. The limits of scRNA-seq include the limited cell number and the involvement of single-cell dissociation procedures, during which protease digestion or heating is required and may lead to cell death or other gene expression changes [44–46]. Recently, another promising transcriptome analysis method, single-nucleus RNA sequencing, has been reported without these deficiencies, which can be used not only in fresh tissue but also in frozen tissue [44] (Table 2).

### 3.2. Cytometry by Time-of-Flight

Cytometry by time-of-flight (CyTOF) or mass cytometry is a variation of flow cytometry in which antibodies are labeled with heavy metal ion tags, rather than fluorochromes. It provides researchers, who seek to explore the mysteries of the immune system, a deeper insight than ever before into the complexity of immune cells. Before the appearance of CyTOF in 2009 at Yale, scientists labeled cellular components with flow cytometry or fluorescence-activated cell sorting (FACS) to collect key cell feature data from a variety of cell phenotypes. The commonly available instruments and reagents determine the limitations of flow cytometry and FACS to analyze at most 20 parameters per cell [8]. In contrast to FACS using fluorescent reporters, CyTOF applies metal isotopes, which are detected via the time-of-flight mass spectrometry technique. It has successfully overcome those limitations and expanded the number of parameters that can be detected simultaneously due to the absence of signal overlap between metal isotopes. Besides, it is translated into four to five times more information, making it particularly suitable for valuable patient samples [47]. Most notably, CyTOF allows an investigator to measure an enormous number of extracellular and intracellular targets simultaneously. This advantage also enables the use of clustering techniques to perform the immunophenotypic classification [48]. However, CyTOF is not as perfect as we expect. Poor resolution for some positive and negative stains, harmful effects on cell division, the large expense associated with the machinery and reagents, lower cell numbers, and longer processing time than flow cytometry [54] are all drawbacks of CyTOF, indicating the areas to be improved upon in the future (Table 2).

Since this technology has been applied to explore the role of natural killer (NK) cells in the immune response to West Nile virus, researchers have started expanding the scope of research from West Nile to multiple sclerosis [55, 56], cancer [57], and systemic autoimmune diseases [58], deepening our understanding of cell biology and human diseases. Recently, this leading technology has been applied to characterize the brain immune system. The unique immune feature of microglia in the human brain compared to peripheral immune cells has been further elucidated by CyTOF. Human brain microglia cells surgically resected from patients with refractory epilepsy exhibited higher levels of the transmembrane marker, TMEM119; the phagocytic receptor, Trem2; and the purinergic receptor, P2Y12, compared with peripheral immune cells obtained from cerebrospinal fluid or blood [59]. These findings depict the region-specific biological profiles of microglia, which may lead to apparently different cell functions. The data parameters generated from such complex cell samples would not be easy to collect using traditional overlapping fluorescent flow cytometry, which indicated the unlimited potential of CyTOF for clinical sample data collection. In the *Nature Medicine* study by Ben-Shaanan et al., CyTOF was used to examine the impact of the reward system activated by the placebo response on immunity [60]. “Designer receptor exclusively activated by designer drugs” (DREADDs) were used to directly activate the dopaminergic neurons in the ventral tegmental area (VTA), simulating the impact of placebo on the reward system. Mouse splenocytes were then isolated for CyTOF after exposure to Escherichia coli. Changes in splenic B cell abundance were successfully obtained by CyTOF, and the results proved that the primary antibacterial immune response and the immune response after pathogen reexposure could be induced by the activation of the reward system. Undoubtedly, CyTOF can deal with complex cellular mixtures. However, the manipulation procedure is very complicated; therefore, detailed instructions about how to perform CyTOF underlying experimentation are needed to achieve data that is reliable and consistent. Currently, a step-by-step experimental protocol to clear the blood cells of the brain was provided by Korin et al. [61]. This method consists of eight major parts, including directions to perfuse the mouse brain, how to dissociate the tissue, and how to configure and run analysis workflows. The comprehensively detailed instructions provided by these authors are expected to assist scientists in conducting research for the foreseeable future.

### 3.3. Whole-Genome Transcriptomic and Proteomic Analysis

Whole-genome transcriptomics and proteomics are well-established approaches that can supply information at the DNA, RNA, and protein levels with outstanding coverage and depth of sequencing. They have played an essential role in genome model development and refinement for a broad spectrum of diseases. During this process, whole-genome transcriptomic and proteomic sequencing analysis improvement significantly promotes rapid progress.

#### 3.3.1. Characterizing the Transcriptional Profile

Ciguatera-induced Chronic Inflammatory Response Syndrome (CIRS) can lead to a variety of neurologic deficits with few therapeutic options for the poor understanding of its pathophysiology. Recently, transcriptional profiles of seven important genes that can distinguish patients from controls have been identified by whole-genome microarrays in whole blood from ciguatera-induced CIRS patients [62]. Besides, this approach is appropriate in studies of vectors of rare infectious diseases. In a previous study, whole-genome sequencing has annotated the transcriptional profiles of Ixodes ricinus ticks and vastly extended the DNA and RNA databases for them, paving the way for further deep study of the vector and its hosts [63].

#### 3.3.2. Clarifying the Genetic Phenotype Heterogeneity

Whole-genome transcriptomic technique was used to explore the deep phenotype of asthmatic patients with IL-17-high immunity by analyzing bronchial biopsies and blood samples [64]. Additionally, malignancies are aggressive with few therapeutic options and poor prognosis. Recently, whole-genome transcriptomic and proteomic sequencing analysis techniques exhibited the potential for clarifying the phenotype heterogeneity in tumors and the underlying mechanisms which promote cancer evolution. Whole-genome transcriptomic analysis has been carried out on surgically resected hepatocellular carcinoma (HCC) samples by a microarray technique for a novel understanding of the tumor microenvironment transcriptional networks [65]. This whole-genome-scale analysis provides a great potential for the actionable strategy design and precision necessary for targeted therapy in cancer treatment.

#### 3.3.3. Identifying Novel Gene Targets and Developing New Antibiotics

Whole-genome transcriptomic and proteomic sequencing analysis was attempted in bacterial studies and the development of new antibiotics, since the first bacterial genome was sequenced twenty years ago. Using this technique, several potential genes and proteins related to glycopeptide resistance were identified by Alexander et al. [66]. Next, Jessica et al. found that the alterations of the bacteria cell surface genes encoded regulatory proteins, which were thought to be a crucial mechanism through which bacteria attained hypervirulence [67]. With these new genome sequencing analysis techniques that have become available, the similarities and differences regarding the genome sequences of Pasteurella multocida have been determined. Regarding the current data, the authors also found an interesting phenomenon, in which no obvious correlation was found between phylogenetic relatedness and host predilection or disease [68]. Hidekazu and colleagues used the whole-genome sequence to profile RS-1, the only strain of Desulfovibrio magneticus bacteria, and revealed the presence of three key genetic components for magnetotactic formation [69]. With respect to antibiotic discovery, approaches based on whole-genome transcriptomic and proteomic sequencing data have yet to identify genes that are important to bacterial survival and growth, thereby revealing potential targets for novel antimicrobial compound development [49].

The advances in mass spectrometry and transcriptome sequencing technologies make the collection of genomic, transcriptomic, and proteomic information from the same tissue sample available. Integration of this data provides a comprehensive and unprecedented insight into the patient's gene profile. In the past year or so, whole-genome transcriptomic and proteomic analysis has been applied in the CNS, combined with an exome sequencing approach to identify critical candidate genes contributing to abnormalities in spine development and cortical neuron dendritic and subsequent neurodevelopmental disorders [50]. With the help of an integrated transcriptomic and proteomic analysis, researchers have validated and improved the present existing annotation of the zebrafish protein-coding genome [51]. Furthermore, several diseases have been investigated at whole genomic, transcriptomic, and proteomic levels, making the therapy strategies more promising. Using these advanced technical tools, multiple candidate genes and signaling pathways potentially involved in Dupuytren's disease have been reported, validated, or proposed, which may help to elucidate the pathogenic mechanisms underlying Dupuytren's disease [52].

Nevertheless, like all other popular areas of research, the current state of whole-genome transcriptomic and proteomic analysis is not without flaws, including the poor sensitivity of the spectrometers, the increased false positives for new peptide hits, and particularly the intrinsic biophysical features that cause some peptides to remain undetectable [53]. In addition, these techniques are extremely dependent on technologies that are used to enable sensitive and large-scale proteome profiling, like the most dominant technology, tandem mass spectrometry (MS/MS) [70]. Another promising technology that they use is ribosomal footprinting, which offers exceptionally valuable information at the translational product level and acts as a bridge between transcriptomics and proteomics data. However, it is technically challenging for many laboratories at present (Table 2).

Although whole-genome transcriptomic and proteomic analysis has been broadly applied in many areas, notably until now, there are few relevant studies found in brain immune research. The reason is likely due to the complexity of the brain structure, making these novel techniques promising in exploring the potential mechanisms of this pathophysiological condition.

## 4. Conclusions and Expectations

To adapt to the environment and fend off external threats, creatures from prokaryotes to humans have strong immune systems, and these immune systems have developed highly specific types of immune cells to prevent and eliminate diseases. The hottest single-cell technology is critical to understanding how the immune system generates potential responses to many different pathogens.

This review summarizes existing novel technologies and discusses their advantages and limitations and explores how these approaches can deepen our understanding of the immune response in neurological diseases. Today, major breakthroughs are scarce in the research of brain injury, including apoptosis [71, 72], inflammation [73, 74], and oxidative stress [75, 76]. Immunology is rapidly gaining attention recently [77]. Additionally, major strides have been made in human science and technology, shedding light on a deep understanding of the immune response after brain injury. However, few neuroscientists have applied these techniques to explore the impact of the immune system on brain repair. It is worth waiting to see how researchers will use these powerful tools to systematically address this issue. To achieve this, information from several fields, such as genomics, transcriptomics, and proteomics at the cellular and organism level, as well as database and computational technologies will be required. Genes and their products do not work independently. Without a doubt, further closer integration of genomics, transcriptomics, proteomics, and relevant promising approaches will be more important than ever before, which can influence the way in which researchers properly understand the brain immune response and, moreover, can sway the way in which other complex neurological problems are solved.

## Figures and Tables

**Table 1:** Comparison of six prominent scRNA-seq protocols. Smart-seq2 is found to be the most sensitive method, and the number of genes detected per cell is the most. Smart-seq is slightly less sensitive than Smart-seq2 and detects almost the same number of genes as Smart-seq2, whereas Drop-seq and MARS-seq have a substantially lower sensitivity and detect considerably fewer genes than others. Interestingly, there are only small differences between these six methods in accuracy and dropout rate. CEL-seq2 is the most powerful method at a sequencing depth of 250,000 reads, but SCRB-seq is the most powerful method with more reads. Drop-seq is the most cost-effective method; SCRB-seq, CEL-seq2, and MARS-seq are less cost-effective, closely followed by Smart-seq and Smart-seq2. Due to its low library cost, Drop-seq may be desirable when analyzing large numbers of cells with low coverage. On the other hand, Drop-seq requires a relatively large number of cells in its current setting. Thus, if few cells are isolated by FACS, SCRB-seq, MARS-seq, or Smartseq2 protocols may be desirable.

Protocol	Sensitivity	Gene number detected	Accuracy	Dropout rates	Power	Cost efficiency	Cell volume
CEL-seq2	More sensitive	More genes	Small differences	Small differences	Most powerful	More cost-effective	Small cells
Drop-seq	Less sensitive	Less genes	Small differences	Small differences	Less powerful	Most cost-effective	Large cells
MARS-seq	Less sensitive	Less genes	Small differences	Small differences	Most powerful	More cost-effective	Fewer cells
SCRB-seq	More sensitive	More genes	Small differences	Small differences	Most powerful	More cost-effective	Fewer cells
Smart-seq	Much more sensitive	Much more genes	Small differences	Small differences	Less powerful	Less cost-effective	Small cells
Smart-seq2	More sensitive	High number of genes	Small differences	Small differences	More powerful	Less cost-effective	Fewer cells

**Table 2:** Methodology of scRNA-seq, CyTOF, and whole-genome transcriptomic and proteomic analysis.

Methods	Molecules analyzed	Parameters	Advantages	Disadvantages	Refs
scRNA-seq	mRNA, surface protein (CITE-seq only)	<20	(1) Incorporates surface protein and mRNA (2) Limited specialized equipment required	(1) Labor-intensive, lowest numbers (2) Protease digestion heating requirement	[43–45]

CyTOF	Protein, glycosylation epitopes, lipids, phosphorylation	>40	(1) Higher simultaneous parameters (2) Permit using clustering to classify cell group (3) Identify cell populations readily without the need to stain with classical markers (4) No spectral overlap and other fluorescence-associated complications (5) Adjust the detector easily	(1) Lower throughput and longer run time than flow cytometry (2) Removal of photometric measurement precludes measurement of forward and side scatter (3) Poor resolution for some stains (4) Cannot isolate live cells (5) Expense of machines and reagents (6) Difficult to detect low abundance antigens (phycoerythrin or allophycocyanin) (7) Ion sensitivity decreases over time	[46–48]

Whole-genome transcriptomic and proteomic analysis	DNA, RNA, protein	>8000 genes (high-quality proteomic data)	Supply information at DNA, RNA, and protein levels	(1) Poor sensitivity of the spectrometers (2) High incorrect incidence for new peptide hits (3) Intrinsic biophysical features that cause some peptides undetectable (4) Largely dependent on computational technologies	[49–53]

## References

[1] Antoine D., Izaskun B., Damien L. (2020). Immune responses and anti-inflammatory strategies in a clinically relevant model of thromboembolic ischemic stroke with reperfusion. *Translational Stroke Research*.

[2] Hidenori S. (2019). Inflammation: a good research target to improve outcomes of poor-grade subarachnoid hemorrhage. *Translational Stroke Research*.

[3] Li L., Devin W. M., Desislava D. (2015). G-CSF attenuates neuroinflammation and stabilizes the blood-brain barrier via the PI3K/Akt/GSK-3*β* signaling pathway following neonatal hypoxia-ischemia in rats. *Experimental Neurology*.

[4] Shao A., Zhu Z., Li L., Zhang S., Zhang J. (2019). Emerging therapeutic targets associated with the immune system in patients with intracerebral haemorrhage (ICH): from mechanisms to translation. *eBioMedicine*.

[5] Shao A., Zhou Y., Yao Y., Zhang W., Zhang J., Deng Y. (2019). The role and therapeutic potential of heat shock proteins in haemorrhagic stroke. *Journal of Cellular and Molecular Medicine*.

[6] Li B., Concepcion K., Meng X., Zhang L. (2017). Brain-immune interactions in perinatal hypoxic-ischemic brain injury. *Progress in Neurobiology*.

[7] Chen Y., Hong-Wei S., Neal E. L. (2019). Single-cell RNA-seq reveals TOX as a key regulator of CD8^+^ T cell persistence in chronic infection. *Nature Immunology*.

[8] James W. T., Kartoosh H., Rabin T. (2007). Modern flow cytometry: a practical approach. *Clinics in Laboratory Medicine*.

[9] Voet S., Srinivasan S., Lamkanfi M., van Loo G. (2019). Inflammasomes in neuroinflammatory and neurodegenerative diseases. *Embo Mol Med*.

[10] Wynn T. A., Chawla A., Pollard J. W. (2013). Macrophage biology in development, homeostasis and disease. *Nature*.

[11] Zmora N., Bashiardes S., Levy M., Elinav E. (2017). The role of the immune system in metabolic health and disease. *Cell Metabolism*.

[12] Saiful I., Una K., Annalena M. (2011). Characterization of the single-cell transcriptional landscape by highly multiplex RNA-seq. *Genome Research*.

[13] Tang F., Barbacioru C., Wang Y. (2009). mRNA-Seq whole-transcriptome analysis of a single cell. *Nature Methods*.

[14] Kohn A. B., Moroz T. P., Barnes J. P., Netherton M., Moroz L. L. (2013). Single-cell semiconductor sequencing. *Methods in Molecular Biology*.

[15] Picelli S., Faridani O. R., Björklund Å. K., Winberg G., Sagasser S., Sandberg R. (2014). Full-length RNA-seq from single cells using Smart-seq2. *Nature Protocols*.

[16] Villani A. C., Satija R., Reynolds G. (2017). Single-cell RNA-seq reveals new types of human blood dendritic cells, monocytes, and progenitors. *Science*.

[17] Josephine B., Gianmarco R. (2019). Single-cell RNA sequencing with Drop-Seq. *Methods in Molecular Biology*.

[18] Jaitin D. A., Kenigsberg E., Keren-Shaul H. (2014). Massively parallel single-cell RNA-Seq for marker-free decomposition of tissues into cell types. *Science*.

[19] Svensson V., Vento-Tormo R., Teichmann S. A. (2018). Exponential scaling of single-cell RNA-seq in the past decade. *Nature Protocols*.

[20] Klein A. M., Mazutis L., Akartuna I. (2015). Droplet barcoding for single-cell transcriptomics applied to embryonic stem cells. *Cell*.

[21] Lun A. T. L., Riesenfeld S., Andrews T., Dao T. P., Gomes T., Marioni J. C. (2019). EmptyDrops: distinguishing cells from empty droplets in droplet-based single-cell RNA sequencing data. *Genome Biology*.

[22] Moon H. S., Je K., Min J. W. (2018). Inertial-ordering-assisted droplet microfluidics for high-throughput single-cell RNA-sequencing. *Lab on a Chip*.

[23] Bose S., Wan Z., Carr A. (2015). Scalable microfluidics for single-cell RNA printing and sequencing. *Genome Biology*.

[24] De Simone M., Rossetti G., Pagani M. (2019). Chromium 10× single-cell 3' mRNA sequencing of tumor-infiltrating lymphocytes. *Methods in Molecular Biology*.

[25] Cao J., Packer J. S., Ramani V. (2017). Comprehensive single-cell transcriptional profiling of a multicellular organism. *Science*.

[26] Sasagawa Y., Danno H., Takada H. (2018). Quartz-Seq2: a high-throughput single-cell RNA-sequencing method that effectively uses limited sequence reads. *Genome Biology*.

[27] Keren-Shaul H., Kenigsberg E., Jaitin D. A. (2019). MARS-seq2.0: an experimental and analytical pipeline for indexed sorting combined with single-cell RNA sequencing. *Nature Protocols*.

[28] Chen X., Teichmann S. A., Meyer K. B. (2018). From tissues to cell types and back: single-cell gene expression analysis of tissue architecture. *Annual Review of Biomedical Data Science*.

[29] Ziegenhain C., Vieth B., Parekh S. (2017). Comparative analysis of single-cell RNA sequencing methods. *Molecular Cell*.

[30] Svensson V., Natarajan K. N., Ly L. H. (2017). Power analysis of single-cell RNA-sequencing experiments. *Nature Methods*.

[31] Haque A., Engel J., Teichmann S. A., Lönnberg T. (2017). A practical guide to single-cell RNA-sequencing for biomedical research and clinical applications. *Genome Medicine*.

[32] Lipniacki T., Paszek P., Brasier A. R., Luxon B. A., Kimmel M. (2006). Stochastic regulation in early immune response. *Biophysical Journal*.

[33] Li Q., Cheng Z., Zhou L. (2019). Developmental heterogeneity of microglia and brain myeloid cells revealed by deep single-cell RNA sequencing. *Neuron*.

[34] Dang J., Tiwari S. K., Agrawal K., Hui H., Qin Y., Rana T. M. (2020). Glial cell diversity and methamphetamine-induced neuroinflammation in human cerebral organoids. *Molecular Psychiatry*.

[35] Song E.-A. C., Min S., Oyelakin A. (2018). Genetic and scRNA-seq analysis reveals distinct cell populations that contribute to salivary gland development and maintenance. *Scientific Reports*.

[36] Silbereis J. C., Pochareddy S., Zhu Y., Li M., Sestan N. (2016). The cellular and molecular landscapes of the developing human central nervous system. *Neuron*.

[37] Quadrato G., Nguyen T., Macosko E. Z. (2017). Cell diversity and network dynamics in photosensitive human brain organoids. *Nature*.

[38] Yoon S. J., Elahi L. S., Pasca A. M. (2019). Reliability of human cortical organoid generation. *Nature Methods*.

[39] Velasco S., Kedaigle A. J., Simmons S. K. (2019). Individual brain organoids reproducibly form cell diversity of the human cerebral cortex. *Nature*.

[40] Welch J. D., Kozareva V., Ferreira A., Vanderburg C., Martin C., Macosko E. Z. (2019). Single-cell multi-omic integration compares and contrasts features of brain cell identity. *Cell*.

[41] Nelson A. C., Mould A. W., Bikoff E. K., Robertson E. J. (2016). Single-cell RNA-seq reveals cell type-specific transcriptional signatures at the maternal-foetal interface during pregnancy. *Nature Communications*.

[42] Dulken B. W., Buckley M. T., Navarro Negredo P. (2019). Single-cell analysis reveals T cell infiltration in old neurogenic niches. *Nature*.

[43] Wester J. C., Mahadevan V., Rhodes C. T. (2019). Neocortical projection neurons instruct inhibitory interneuron circuit development in a lineage-dependent manner. *Neuron*.

[44] Kaya J. E. M., Anupama S., Kory R. J., Michael C. K., Matthew W. K., Ariel J. L. (2018). Isolation of adult spinal cord nuclei for massively parallel single-nucleus RNA sequencing. *Journal of Visualized Experiments*.

[45] Stubbington M. J. T., Rozenblatt-Rosen O., Regev A., Teichmann S. A. (2017). Single-cell transcriptomics to explore the immune system in health and disease. *Science*.

[46] Higdon L. E., Schaffert S., Khatri P., Maltzman J. S. (2019). Single cell immune profiling in transplantation research. *American Journal of Transplantation*.

[47] Behbehani G. K. (2017). Applications of mass cytometry in clinical medicine: the promise and perils of clinical CyTOF. *Clinics in Laboratory Medicine*.

[48] Sean C. B., Erin F. S., Peng Q. (2011). Single-cell mass cytometry of differential immune and drug responses across a human hematopoietic continuum. *Science*.

[49] Fields F. R., Lee S. W., McConnell M. J. (2017). Using bacterial genomes and essential genes for the development of new antibiotics. *Biochemical Pharmacology*.

[50] Uddin M., Unda B. K., Kwan V. (2018). *OTUD7A* Regulates Neurodevelopmental Phenotypes in the 15q13.3 Microdeletion Syndrome. *American Journal of Human Genetics*.

[51] Kelkar D. S., Provost E., Chaerkady R. (2014). Annotation of the Zebrafish Genome through an Integrated Transcriptomic and Proteomic Analysis. *Molecular & Cellular Proteomics*.

[52] Shih B., Watson S., Bayat A. (2012). Whole genome and global expression profiling of Dupuytren's disease: systematic review of current findings and future perspectives. *Annals of the Rheumatic Diseases*.

[53] Dimitrakopoulos L., Prassas I., Diamandis E. P. (2016). Proteogenomics: opportunities and caveats. *Clinical Chemistry*.

[54] Yao Y., Liu R., Shin M. S. (2014). CyTOF supports efficient detection of immune cell subsets from small samples. *Journal of Immunological Methods*.

[55] Cole J. E., Park I., Ahern D. J. (2018). Immune cell census in murine atherosclerosis: cytometry by time of flight illuminates vascular myeloid cell diversity. *Cardiovascular Research*.

[56] Winkels H., Ehinger E., Vassallo M. (2018). Atlas of the immune cell repertoire in mouse atherosclerosis defined by single-cell RNA-sequencing and mass cytometry. *Circulation Research*.

[57] Kourelis T. V., Villasboas J. C., Jessen E. (2019). Mass cytometry dissects T cell heterogeneity in the immune tumor microenvironment of common dysproteinemias at diagnosis and after first line therapies. *Blood Cancer Journal*.

[58] der Kroef M., den Hoogen L. L., Mertens J. S. (2020). Cytometry by time of flight identifies distinct signatures in patients with systemic sclerosis, systemic lupus erythematosus and Sjögrens syndrome. *European Journal of Immunology*.

[59] Brewster A. L. (2019). Human microglia seize the chance to be different. *Epilepsy Currents*.

[60] Ben-Shaanan T. L., Azulay-Debby H., Dubovik T. (2016). Activation of the reward system boosts innate and adaptive immunity. *Nature Medicine*.

[61] Korin B., Dubovik T., Rolls A. (2018). Mass cytometry analysis of immune cells in the brain. *Nature Protocols*.

[62] Ryan J. C., Wu Q., Shoemaker R. C. (2015). Transcriptomic signatures in whole blood of patients who acquire a chronic inflammatory response syndrome (CIRS) following an exposure to the marine toxin ciguatoxin. *BMC Medical Genomics*.

[63] Cramaro W. J., Revets D., Hunewald O. E., Sinner R., Reye A. L., Muller C. P. (2015). Integration of Ixodes ricinus genome sequencing with transcriptome and proteome annotation of the naïve midgut. *BMC Genomics*.

[64] Östling J., van Geest M., Schofield J. P. R. (2019). IL-17-high asthma with features of a psoriasis immunophenotype. *Journal of Allergy and Clinical Immunology*.

[65] Tanaka S. (2018). Precision medicine based on surgical oncology in the era of genome-scale analysis and genome editing technology. *Annals of Gastroenterological Surgery*.

[66] Scherl A., François P., Charbonnier Y. (2006). Exploring glycopeptide-resistance in Staphylococcus aureus: a combined proteomics and transcriptomics approach for the identification of resistance-related markers. *BMC Genomics*.

[67] Galloway-Peña J., DebRoy S., Brumlow C. (2018). Hypervirulent group a streptococcus emergence in an acaspular background is associated with marked remodeling of the bacterial cell surface. *PLoS One*.

[68] Boyce J. D., Seemann T., Adler B., Harper M. (2012). Pathogenomics of Pasteurella multocida. *Current Topics in Microbiology and Immunology*.

[69] Nakazawa H., Arakaki A., Narita-Yamada S. (2009). Whole genome sequence of Desulfovibrio magneticus strain RS-1 revealed common gene clusters in magnetotactic bacteria. *Genome Research*.

[70] Prasad T. S., Mohanty A. K., Kumar M. (2017). Integrating transcriptomic and proteomic data for accurate assembly and annotation of genomes. *Genome Research*.

[71] Shao A., Wang Z., Wu H. (2016). Enhancement of autophagy by histone deacetylase inhibitor trichostatin A ameliorates neuronal apoptosis after subarachnoid hemorrhage in rats. *Molecular Neurobiology*.

[72] Wang Z., Zhou F., Dou Y. (2018). Melatonin alleviates intracerebral hemorrhage-induced secondary brain injury in rats via suppressing apoptosis, inflammation, oxidative stress, DNA damage, and mitochondria injury. *Translational Stroke Research*.

[73] Shao A., Wu H., Hong Y. (2016). Hydrogen-rich saline attenuated subarachnoid hemorrhage-induced early brain injury in rats by suppressing inflammatory response: possible involvement of NF-*κ*B pathway and NLRP3 inflammasome. *Molecular Neurobiology*.

[74] Suzuki H. (2019). Inflammation: a good research target to improve outcomes of poor-grade subarachnoid hemorrhage. *Translational Stroke Research*.

[75] Xu W., Li T., Gao L. (2019). Sodium benzoate attenuates secondary brain injury by inhibiting neuronal apoptosis and reducing mitochondria-mediated oxidative stress in a rat model of intracerebral hemorrhage: possible involvement of DJ-1/Akt/IKK/NF*κ*B pathway. *Frontiers in Molecular Neuroscience*.

[76] Fumoto T., Naraoka M., Katagai T., Li Y., Shimamura N., Ohkuma H. (2019). The role of oxidative stress in microvascular disturbances after experimental subarachnoid hemorrhage. *Translational Stroke Research*.

[77] Tong L. S., Shao A. W., Ou Y. B. (2017). Recombinant Gas 6 augments Axl and facilitates immune restoration in an intracerebral hemorrhage mouse model. *Journal of Cerebral Blood Flow and Metabolism*.

